# Remote workers’ free associations with working from home during the COVID-19 pandemic in Austria: The interaction between children and gender

**DOI:** 10.3389/fpsyg.2022.859020

**Published:** 2022-08-04

**Authors:** Martina Hartner-Tiefenthaler, Eva Zedlacher, Tarek Josef el Sehity

**Affiliations:** ^1^Labor Science and Organization, Institute of Management Science, TU Wien, Vienna, Austria; ^2^Department of Business and Management, Webster Vienna Private University, Vienna, Austria; ^3^Institute of Business and Economic Psychology, Faculty of Psychology, Sigmund Freud Private University, Vienna, Austria; ^4^Institute of Cognitive Sciences and Technologies, Italian National Research Council, Rome, Italy

**Keywords:** children, gender, boundary management strategies, non-work interrupting work behaviors, working from home (WFH), telework, time-spatial flexibility, free association technique

## Abstract

Empirical evidence from the COVID-19 pandemic shows that women carried the major burden of additional housework in families. In a mixed-methods study, we investigate female and male remote workers’ experiences of working from home (WFH) during the pandemic. We used the free association technique to uncover remote workers’ representations about WFH (i.e., workers’ reflection of subjective experiences). Based on a sample of 283 Austrian remote workers cohabitating with their intimate partners our findings revealed that in line with traditional social roles, men and women in parent roles are likely to experience WFH differently: Mothers’ representations about WFH emphasize perceived incompatibility between the work and non-work sphere whereas fathers’ representations highlight work-family facilitation of WFH. However, gender differences were also prevalent for women and men without children: Women seem to particularly benefit from more concentration at home, whereas men consider WFH as more efficient, practical and leading to less work. Thus, our findings imply that gender affected perceptions of WFH during the pandemic independently from children, but children seemed to increase the existing burden, in particular for women. To conclude, WFH can generally be seen as an enabler to reduce work-life/family conflict for both women and men, but bears different challenges based on the contextual (family) situation.

## Introduction

The COVID-19 pandemic has caused drastic disruptions on the labor market and led to an increase in alternative work arrangements such as short or remote work (Chung et al., 2021; Yavorsky et al., 2021). During the early pandemic in 2020, about 30 to 40 percent of the total workforce were *working from home* (WFH) (Eurofound, 2020; Statistik Austria, 2020) compared to only 13 percent in Austria prior to the pandemic in 2015 (Bock-Schappelwein, 2020). Parents were particularly challenged to cope with the additional demands as schools were closed during the lockdown. Research collected during this time indicates that mothers were disproportionately taking on the new family demands. They did so often in addition or at the expense of hours in their work sphere (Collins et al., 2021; Derndorfer et al., 2021; Çoban, 2022), although men in egalitarian households also increased their share of domestic (Chung et al., 2021; Shockley et al., 2021).

However, studies from the pandemic have often focused on investigating the experiences of men and women *with* children only (e.g., Sevilla and Smith, 2020; Chung et al., 2021; Collins et al., 2021; Mangiavacchi et al., 2021; Petts et al., 2021; Çoban, 2022) and/or investigated additional (family) task demands and distribution of housework or working hours before and during the pandemic (e.g., Derndorfer et al., 2021; Hipp and Bünning, 2021; Zamarro and Prados, 2021). In our study, we particularly investigate the effect of children and gender. In times of crises, traditional social roles and ideal norms of fathers as “breadwinners” and mothers as “caregivers” (Eagly and Wood, 2012) might re-emerge when there are dependent children in the same household. Consequently, mothers working from home might face more interruptions than men as well as distress due to a perceived incompatibility of roles when “family calls.” However, social roles and ideal worker images are dynamic and evolve (Lott and Klenner, 2018), yet in-depth research on working fathers’ practices in WFH during the pandemic remains scarce.

Hence, the overall aim of this study is to investigate whether WFH was a gendered experience for women and men during the pandemic per se, or whether the existence of children in the same household made women more than men juggle additional demands from the family sphere during WFH. Thus, this paper tackles the following research question: *Do representations about WFH differ between women and men, considering their roles as fathers and mothers*?

We build our hypotheses on the boundary management literature, and the assumption of a (gendered) separation and incompatible demands between the “public” vs. the “family” sphere. Traditionally, WFH has been considered as a working arrangement that encompasses the privilege to autonomously decide upon the workplace (Allen et al., 2021). Often women chose WFH as the (only) way to juggle work and family demands (Sullivan and Lewis, 2001). In 2020, governmental restrictions due to the pandemic have forced both women and men to adjust their private environment and let work move into private homes. Parents were no longer able to outsource many activities such as housework, childcare or schooling. When establishing an office in the private home, traditional physical and temporal boundaries between one’s work and non-work sphere became blurred (in this paper, we used “non-work” sphere as a synonym for all non-work demands including family and other non-work activities). Remote workers might be more often interrupted by “uncontrollable” demands from the non-work sphere, in particular if they have children and there is no time or space to establish physical boundaries at home (e.g., a separate office room).

Using an open explorative approach with the free association technique (el Sehity and Kirchler, 2006; Joffe and Elsey, 2014), we investigate participants’ representations about WFH and analyze underlying (gendered) notions. Representations cover the subjective realm of ideas, values and practices that people form and hold of their world (el Sehity and Kirchler, 2006). Comparing free associations towards WFH between women and men (with/without children) enables us to investigate participants’ representations about WFH and sheds light on the different subjective meanings of WFH as well as underlying (gendered) experiences and expectations.

Based on this, our study makes the following contributions to theory and practice.

First, we used an approach that allows us to combine elements from both qualitative and quantitative research to further the understanding of the participants’ work and non-work sphere. Using this technique (el Sehity and Kirchler, 2006; Joffe and Elsey, 2014), we collected free verbal associations (Nelson et al., 2000) towards the stimulus “working from home” from workers who had experience with WFH during the first lockdown in Austria. After writing down their associations, each worker evaluated each association whether they considered it as negative, neutral or positive. This approach has been successfully applied in social psychology (e.g., Guimelli, 1993; Dany et al., 2015), personality research (e.g., Kuška et al., 2016), and economic psychology (e.g., Zehnter and Kirchler, 2020). Free associations evoked by symbolic stimuli express a person’s experiences related to the stimulus and / or her mental attitudes, such as values and ideas towards the stimulus. These associations provide valuable insights into a person’s subjective experience of the stimulus without imposing biased rationalization processes, for instance, when narrated in interviews (Tadajewski, 2006). In contrast to survey questions, this form of data collection does not steer participants’ thoughts and allows freedom of expression (Kulich et al., 2005; Zehnter and Kirchler, 2020). It elicits default thinking (Rozin et al., 2002) and enables us to investigate gendered assumptions about WFH by comparing associations produced by female and male workers (with/without children). Therefore, free associations are considered ecologically valid (Joffe and Elsey, 2014).

Second, we echo Russo et al. (2018) call that the (family) context needs to be investigated to better understand boundary management strategies between work and non-work. Allen et al. (2021) considered other household members for researching boundary management strategies and work-life/family balance, but the interaction between gender and dependent children still needs to be explored systematically. In this study, we shed light on the interaction between gender and children and investigate how under-researched groups such as women and men without children as well as men with children enact or interrupt the spheres of work and non-work while working from home.

Third, our findings also contribute to practice. Traditionally, WFH was established for improving the reconcilability for women as predominant caretakers for children (Allen et al., 2021). Our findings indicate that organizations seem to have neglected the role of men for childcare duties as fathers significantly more often criticized the limited access to WFH. To initiate a deep-level change, organizations need to enable men with children to take over these tasks in families. Thus, employers need to be more aware of the different situations of workers when providing access to WFH. Further, despite workers’ appreciation of flexibility, WFH bears a flexibility paradox (Chung, 2022) reflected in self-exploitation due to the challenge of blurred boundaries between work and non-work. This paradox is more prevalent among mothers and therefore needs to be actively addressed by the organizations to enable workers to segment work from non-work also when working from home. Interestingly, gender differences also emerged for women and men without children: Women primarily emphasized the benefit for the work sphere (i.e., more concentration) whereas men emphasized the gain for the non-work sphere (i.e., efficient and less work). These associations suggest that organizations need to build awareness about existing inequalities including the (physical) work sphere.

### (Gendered) boundaries between work and non-work spheres

Boundaries between work and non-work have been described in boundary theory (Ashforth et al., 2000) and work-family border theory (Clark, 2000). They are social constructions which can be strengthened *via* specific temporal or spatial demarcations (e.g., a change of clothes or space), and are relatively stable and durable (Ammons, 2013). When working from home, the physical demarcations between work and non-work such as separate office spaces or changing of clothes after work might have become less prevalent (Delanoeije et al., 2019; Kerman et al., 2021).

Generally, the masculine-biased image of the ideal worker represents workers who can fully focus on the work sphere and have no domestic or care obligations to worry about during and after work, i.e., resembling the male breadwinner model (Acker, 1990; Rothbard and Ollier-Malaterre, 2016; Kossek et al., 2021). Social role theory (Eagly and Wood, 2016) suggests that women (should) act as caregivers, engage in (family) relationships and answer to family demands (Eagly and Wood, 2016; Nsair and Piszczek, 2021). When employed, mothers also have to comply with the ideal worker image despite a pressure to uphold or even prioritize demands from the non-work sphere (see Russo et al., 2018). However, fathers often are solely subjected to the ideal worker image and view their engagement in the non-work sphere as privilege and individual choice rather than as entitlement or duty (e.g., Sullivan and Lewis, 2001; Borgkvist et al., 2018). Halford (2006) showed that fathers working from home found ways to separate work and family spheres in the domestic space either spatially or psychologically, often with their female partner acting as “police” to ensure that the boundaries were respected by all (dependent) family members. As such, fathers considered WFH as a way to be closer to the family while they only had to fulfill family demands at a sub-standard level, and could still prioritize the work sphere (Halford, 2006).

Although the pure male breadwinner model has been subject to change and modernization (Lott and Klenner, 2018), Austria is still characterized as a country with very traditional gender roles and highly gendered separation of work vs. non-work spheres (e.g., Buber-Ennser, 2015; Derndorfer et al., 2021). A recent qualitative study by Schmidt (2021) among Austrian employers and workforce members showed that respondents construct part-time and flexible work mainly as an option for women with (potential) children, but not for men. Indeed, working mothers commonly reduce working hours substantially as long as their child is dependent and/or at least reaches school age (Buber-Ennser, 2015; OECD, 2015).

### Working from home and blurred boundaries

WFH is used synonymously with telecommuting, telework or remote work. It encompasses workers’ choice to decide upon the place of work, which is often accompanied with the choice of working time (Allen et al., 2015; Wessels et al., 2019). It bears several advantages such as saving commuting time. When fewer workers travel to work, traffic is also decreased which helps to protect the environment (Tomei, 2021). Furthermore, it relates to greater job satisfaction due to workers’ increased autonomy (Gajendran and Harrison, 2007), less work-role stress (Gajendran and Harrison, 2007) and less work exhaustion (Sardeshmukh et al., 2012). In contrast, negative aspects of WFH are potential distractions from the non-work sphere bearing the threat of procrastination (Allen et al., 2015) as well as social isolation (Golden et al., 2008). Moreover, once workers have (mobile) devices or other communication technologies available for WFH, it might also increase their frequency to access work even after working hours. When the boundary between work and non-work is blurred, well-being is potentially impaired due to extended availability for work (Dettmers et al., 2016; Schlachter et al., 2018).

Traditionally, WFH has been considered to facilitate role transitions and enable (female) workers to better manage boundaries between work and family demands and as such increase work-life/family balance and reduce perceived *work-family conflict* (Allen et al., 2015; Chung and van der Lippe, 2020). Work-family conflict is defined as an accumulation of daily events, where incompatible demands between work and family roles impair participation in both roles (Hunter et al., 2019). Empirical results, however, show limited beneficial effects of WFH on reducing work-family conflict (e.g., Rau and Hyland, 2002; Allen et al., 2015), although there is evidence for so-called *work-family facilitation* in family settings with small children (see Chung and van der Lippe, 2020). Chung et al. (2021) argue that mothers associate WFH rather with the possibility to integrate or prioritize (intense) family demands into their existing work schedule. Fathers’ motivation to work remotely is often due to a need for flexibility and autonomy and/or the possibility to work longer hours; motives, which do not challenge the ideal worker image (Sullivan and Lewis, 2001).

### Managing boundaries during the pandemic

We assume that boundaries between work and non-work are managed differently when there are dependent children in the same household. As external childcare facilities had to close abruptly (due to governmentally enforced lockdowns), working parents might have experienced (family) interruptions requiring immediate attention and resulting in frequent role transitions. Hence, tasks that had been outsourced before (e.g., childcare, cleaning, etc.) had to be integrated into daily family life and placed additional (boundary management) demands on remote workers. Some workers had little experience as well as time to prepare and organize (additional) family demands. Thus, it is likely that working parents were particularly challenged to manage their boundaries between work and family which became increasingly blurred and partly even disappeared (see for example the study by Otonkorpi-Lehtoranta et al., 2021).

The pandemic might have led to more frequent role transitions between work and family as interruptions from the non-work sphere increased when there were children living in the same household. Hence, we assume that workers who also had the role of parents had more permeable boundaries in their work sphere than workers without dependent children.

**Hypothesis 1:** Workers with children in the same household interrupt their work for non-work demands significantly more than workers without children in the same household.

Although fathers also increased their share in childcare during the early lockdown (Adams-Prassl et al., 2020; Shafer et al., 2020, Chung et al., 2021; Mangiavacchi et al., 2021), they often did so to a lesser degree than mothers (Sevilla and Smith, 2020; Mangiavacchi et al., 2021; Petts et al., 2021), in particular when they have not equally shared unpaid work prior to the lockdown (Chung et al., 2021; Shockley et al., 2021) or earned substantially more than their female partners (Mangiavacchi et al., 2021). Thus, we assume that mothers might not only face more demands from the non-work sphere than fathers due to the existing social role pressures, but they are also more likely than men to perceive an incompatibility of roles from the two spheres and may then strive to protect the non-work sphere at the expense of the work sphere. Literature shows that women let work interfere with family demands more than men (Innstrand et al., 2009; Shockley et al., 2017; Nsair and Piszczek, 2021), and this might have even increased during the pandemic due to the (new) additional tasks in the non-work sphere (Derndorfer et al., 2021). Therefore, we assume:

**Hypothesis 2:** Female workers with children in the same household interrupt their work for non-work demands significantly more than male workers with children in the same household.

### Representations about working from home during the pandemic

Although generally, WFH reduces job-related negative affect and increases positive job-related affect (Anderson et al., 2015), the evaluation might have been different during the COVID-19 pandemic. During the lockdown, WFH was often established involuntarily, i.e., workers had to stay home to avoid contagion with the virus. In order to explore women’s and men’s experiences of WFH during the pandemic and capturing their representations (el Sehity and Kirchler, 2006), we use free associations towards the stimulus “working from home” and compare the valence (i.e., averaged number of positive associations per participant) of the produced semantic content.

Overall, WFH was connected to coping with new work demands such as the use of new virtual work tools for communication and collaboration during the pandemic (Allen et al., 2021). Workers had to cope with challenges for managing the boundaries between work and family. Parenthood was found to be a main driver for lower job satisfaction during and after the early lockdown in Germany (Hipp and Bünning, 2021). However, literature lacks knowledge about how the non-work sphere influences attitudes about WFH. Even though WFH is generally seen to be beneficial for reconcilability between work and family demands (ten Brummelhuis and van der Lippe, 2010; Chung and van der Lippe, 2020), we assume that workers with children are more challenged than workers without children and thus, have a less positive attitude towards WFH. Thus, we hypothesize:

**Hypothesis 3:** Workers with children in the same household experience WFH less positively than workers without children in the same household.

New tasks such as home schooling or non-routine childcare might foster gendered role assignments (Emslie and Hunt, 2009). A recent study showed a shift towards the extremes: While more men contributed to housework and childcare due to the pandemic, it was also more women who had to take on the main responsibility for housework and childcare (Hank and Steinbach, 2021). In a representative study among couples who both worked from home, 80% of mothers, but only 50% of fathers engaged in home schooling during the pandemic (Dunatchik et al., 2021). In line with this, mothers perceived their productivity to be lower after the first lockdown (Carroll et al., 2020), which might also affect their experiences with WFH. Thus, we assume for our last hypothesis:

**Hypothesis 4:** Female workers with children in the same household experience WFH less positively than male workers with children in the same household.

## Materials and methods

### Procedure

We collected data from June 26th until October 25th, 2020. This was a period where participants had previously experienced a lockdown due to COVID-19, but no restrictions were in place during the time of data collection. To better understand the context of our study we briefly summarize the lockdown situation prior to our data collection. Similar to other countries, governmental measures were implemented in Spring 2020 in Austria to reduce the high infection rates of COVID-19. Many businesses were required to close their offices and – where possible – enable their workers to work from home. Citizens were asked to stay home if one of the following four reasons did not apply (Bundesministerium für Digitalisierung und Wirtschaftsstandort, 2020):

(1) having to go to work in case WFH is not possible, which is applicable for workers from areas such as police, medical staff, delivery, childcare, etc.
(2) having to run absolutely essential errands such as grocery shopping or going to the pharmacy.
(3) helping other people in Austria to run absolutely essential errands for them because they are not able to do them themselves.
(4) being outside such as going for walks is only allowed alone or with people who live in the same home.

These restrictions were in place in Austria from 16th March until 1st May 2020 and resulted in a high prevalence of WFH among office workers. To further shed light on their situation, our study focuses on workers who were able to work from home during this lockdown (although we collected our data after this first lockdown).

For data collection, we randomly selected approximately 10,000 workers from the members’ list of the Chamber of Labor from Lower Austria (note: in Austria, workers have obligatory membership in the Chamber of Labor). The Chamber of Labor invited selected workers to participate in our study *via* a postcard sent to their homes providing the link to our online survey. Our goal was to attract a more diverse sample of participants since women and participants with higher educational degrees are often over-represented (e.g., Langenkamp et al., 2022). However, we are aware that self-selection bias is still likely (Søgaard et al., 2004). In addition to the postcards, we used mailing lists to attract even more participants for our study. Inspecting the data, we found no differences between the data collected *via* the postcards and *via* the mailing lists.

Since living arrangements were decisive about how workers experienced the lockdown during the pandemic (e.g., single parents and individuals living alone had a higher risk of experiencing loneliness and care-related worries; Langenkamp et al., 2022), we focused only on workers who lived with their intimate partners (with and without children) in the same household.

### Sample

From a sample of 601 workers, 299 data sets fulfilled the two defined criteria: (1) living with the intimate partner in the same household and (2) working from home at least during the first lockdown. Sixteen further cases had to be excluded due to missing information on gender or free associations. The final sample encompassed data of 283 participants.

In total, 46.4 % of the participants were female and 46.3 % had at least one child living in their household. Participants’ average age was 41.1 years (*SD* = 10.6). Of the total sample, 23.3% held a leadership position and, on average, the participants worked 35.0 (*SD* = 14.6) hours / week including overtime. Table 1 gives an overview of socio-demographic differences between the female and male sample. It reflects the gendered job segregation in Austria as men are more likely to hold a leadership position and rather work in the information and communication industry, whereas women are more likely to work in retail (Statistik Austria, 2022).

**TABLE 1:** Socio-demographics of participants (*N* = 283).

	Women (*n* = 129, 46.4%)	Men (*n* = 154, 53.6%)
**Socio-demographics**		
Age *t*(281) = 3.15, *p* = 0.002, *d* = 10.38	*M* = 38.89; *SD* = 9.80	M = 42.80; *SD* = 10.84
Education	*χ2*(3, 283) = 0.64, *p* = 0.886	
Obligatory school or apprenticeship	16 (12.4%)	21 (13.6%)
Vocational/technical school	9 (7.0%)	13 (8.4%)
High School Diploma	43 (33.3%)	54 (35.1%)
University/Polytechnic degrees	61 (47.3%)	66 (42.9%)
Family situation and household conditions*		
Number of persons living in the household *t*(281) = 2.926, *p* = 0.004, *d* = 0.34	*M* = 2.76, *SD* = 1.08 *Md* = 2.00;	*M* = 3.13, *SD* = 1.12 *Md* = 3.00;
Workers with youngest child (age group) in their household	*χ2*(5, 283) = 22.94, *p* < 0.001	
< 3 years*	4*	29
3 to < 6 years	13	9
6 to < 10 years	8	9
10 to < 14 years	8	11
> = 14 years	17	28
Workers with children in household	47 (36.4%)	84 (54.5%)
Workers without children in household	82 (63.6%)	70 (45.5%)
Square meters of your flat/house: t(279) = 0.713, *p* = 0.476	*M* = 113.51; *SD* = 53.30	*M* = 117.79; *SD* = 47.30
Commuting time (in minutes) t(280) = 1.907, *p* = 0.58;	*M* = 30.47; *SD* = 23.61	*M* = 36.21 *SD* = 26.40
**Employment characteristics**		
Employment status	*χ2*(1, 283) = 0.419, *p* = 0.517	
Employed and in short-time work	19 (14.1%)	18 (11.5%)
Employed and *not* in short-time work	110 (85.9%)	136 (88.5%)
What industry are you in?	*χ2*(9, 283) = 17.55, *p* = 0.041	
Energy and water supply; sewage and waste disposal	6 (4.7%)	8 (5.2%)
Information and Communication	18 (14.1%)	44 (28.6%)
Education and Instruction	7 (5.5%)	3 (1.9%)
Research and Development	15 (11.7%)	17 (11.0%)
Advertising and Marketing	16 (12.5%)	10 (6.5%)
Wholesale and retail trade; repair of motor vehicles and motorcycles	14 (10.9%)	7 (4.5%)
Provision of financial and insurance services	12 (9.4%)	14 (9.1%)
Professional, scientific and technical service activities	6 (4.7%)	13 (8.4%)
Management Consulting, Management and Leadership	3 (2.3%)	4 (2.6%)
Other	31 (24.2%)	34 (22.1%)
Leadership role	*χ2*(1, 283) = 11.63, *p* = 0.001	
Yes	18 (14.0%)	48 (31.2%)
No	111 (86.0%)	106 (68.8%)
**Employer characteristics**		
Size of the organization	*χ2*(3, 283) = 8.943, *p* = 0.030	
= < 10 workers	25 (19.4%)	13 (8.4%)
11-50 workers	30 (23.3%)	38 (24.70%)
51-250 workers	19 (14.7%)	30 (19.5%)
more than 250 workers	55 (42.6%)	73 (47.4%)
Number of persons in team?	*χ2*(4, 283) = 4.382, *p* = 0.357	
Works alone (0)	19 (17.7%)	14 (9,1%)
Duo-team (1)	8 (6.2%)	11 (7.1%)
Small teams (2-5)	64 (49.6%)	71 (46.1%)
Mid teams (6-12)	34 (26.4%)	48 (31.2%)
Big teams (13-30)	4 (3.1%)	10 (6.5%)

*The grossly underrepresented group of female workers with children aged less than three years is likely to be related to the fact that up to three years of maternity-leave are granted after birth - which is traditionally mainly claimed by mothers in Austria.

### Measures

The online survey captured questions about the current and previous work situation including the location of work during the first lockdown and socio-demographics. For the purpose of this study, we focused on the content associated with “working from home”, which was assessed at the start of the survey. Additionally, we analyzed the evaluation scores of the associations and participants’ reported boundary management strategies.

*Free associations with “working from home”:* To obtain information about workers’ representations we used free verbal associations. In more detail, we asked the participants to associate to the stimulus “working from home” (in German: “*Arbeit von zuhause*”). Participants could write up to five free associations towards this stimulus. We then asked participants to assess the valence of each of their indicated association (positive, neutral, or negative). Participants’ evaluation of the content expresses the attitude towards the associated content and is considered a proxy of a person’s attitude towards the stimulus (el Sehity and Kirchler, 2006). For our analysis we focused on the number of positive associations per participant.

*Boundary management strategies* capture workers’ approach to demarcate their boundary between work and non-work spheres by assessing how often they interrupt their non-work activities due to work issues or vice versa. For measuring, we used the work-life indicator scale (Kossek et al., 2012) and asked four items for non-work interrupting work behaviors (sample item: “I allow work to interrupt me when I spend time with my family or friends.”). Since the original scale was published in English (Kossek et al., 2012), we used Brislin’s (1970) back-translation method for translating the items. Response options of the four items ranged from never (1) to always (7). Reliability was satisfactory with α = 0.80.

Finally, various variables capturing *socio-demographics* (e.g., age, gender, etc.) and information about the *work characteristics* (e.g., leadership position, working hours, etc.) were asked.

*Category development: Content of the associations.* To capture the content of the associations we inductively categorized 1168 associations produced by 283 participants. On average 4.08 associations (*SD* = 1.17) were verbalized per participant. Of these 1168 free associations 754 different terms emerged and were categorized using a data-driven inductive approach to capture the most salient dimensions associated to WFH. With the help and discussion with the other authors and research assistants, the first author inductively created 29 mutually exclusive categories based on the content of the associations (not taking the evaluations into account) and clustered them to the following ten superordinate categories (the category system is described in the Supplementary material): (1) meaning of WFH and COVID-19, (2) personal experience with WFH, (3) requirements and conditions at home, (4/5) positive/negative attitudes about WFH, (6/7) positive/negative consequences for working, (8/9) positive/negative consequences for private life or health, and (10) positive consequences for society. In a second step, an independent rater, who was naïve to the research questions, assigned all associations to these 29 categories. Prior to this task, the meaning of each category was discussed in depth, and anchor examples were provided. Based on the initial categorization, the inter-rater agreement was satisfactory with *Cohen’s Kappa* = 0.85. Subsequently, the two raters discussed the associations, which they had categorized differently, in order to reach full agreement between the two raters.

### Quantitative analysis: Testing hypotheses

For hypothesis 1 we tested whether workers with children interrupt their work for non-work demands significantly more frequently than workers without children in the same household. Non-work to work interruptions of participants with and without children were normally distributed, as assessed by Shapiro-Wilk’s test (*p* > 0.05). Also, there were no outliers in the data and homogeneity of variances was achieved for both groups assessed by Levene’s test for equality of variances (*p* = 0.807). Testing the hypothesis with one-way ANCOVA, controlling for age (*F* (1, 275) = 6.69, *p* = 0.010, partial η2 = 0.02), actual working hours (*F* (1, 275) = 0.88, *p* = 0.348), leadership role (*F* (1, 275) = 9.05, *p* = 0.003, partial η2 = 0.03), and gender (*F* (1, 275) = 8.50, *p* = 0.004, partial η2 = 0.03) we could not find a difference between workers with children (*M* = 3.67, *SE* = 0.11) and without children (*M* = 3.83, *SE* = 0.11). Thus, we maintain the null hypothesis (*F* (1, 275) = 1.02, *p* = 0.315).

For hypothesis 2 we tested whether female workers with children interrupt their work for family demands significantly more than male workers with children in the same household. We analyzed non-work to work interruptions on the subset of workers with children in their households (*n* = 131) and tested whether the scores were higher for female workers. Also here, homogeneity of variances was achieved for both groups assessed by Levene’s test for equality of variances (*p* = 0.823). Testing the hypothesis with one-way ANCOVA, controlling for age (*F* (1, 126) = 3.00, *p* = 0.086), actual working hours (*F* (1, 126) = 0.02, *p* = 0.902), and leadership role (*F* (1, 126) = 3.73, *p* = 0.056), we found no significant difference between female workers with children (*M* = 3.45, *SE* = 0.21) and male workers with children (*M* = 3.85, *SE* = 0.15) and thus maintain the null hypothesis (*F* (1, 126) = 3.27, *p* = 0.160).

Subsequently, we tested whether workers with children in the same household experience WFH less often positively than workers without children in the same household (Hypothesis 3). The number of positively evaluated associations was calculated per person resulting in a minimum score of 0 for those who evaluated none of their free associations positively and a maximum of 5 for those who evaluated all of their free associations to WFH positively.

**FIGURE 1:**
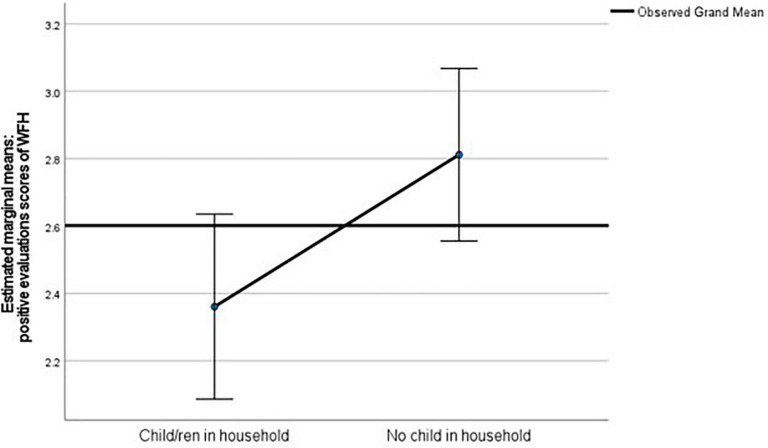
Line chart of estimated marginal means of number of positive evaluations to WFH (min. = 0 - max. = 5) per household (HH) condition. Covariates appearing in the model are evaluated at the following values: Age = 41.08, Leadership position = 0.23. Current contractual working hours per week = 39.13, Female = 0.46; error bars: 95% CI.

Although positive evaluations scores of workers were not normally distributed, as assessed by Shapiro-Wilk’s test (*p* > 0.05), due to our sample size (*N* > 30) the central limit theorem applies for the use of a parametric test (e.g., Kwak and Kim, 2017). Homogeneity of variances applies for all groups, as assessed by Levene’s test for equality of variances (*p* = 0.266). A one-way ANCOVA was used to control for age (*F* (1, 275) = 0.15, *p* = 0.696), actual working hours (*F* (1, 275) = 0.51, *p* = 0.48), leadership role (*F* (1, 275) = 1.51, *p* = 0.220), and gender (*F* (1, 275) = 0.69, *p* = 0.408) to test if workers with children at home evaluated their associations to WFH less positively than workers without children at home. The group difference −0.43, 95% CI [−0.81, −0.04] was statistically significant, *F* (1, 275) = 4.72, 1-sided *p* = 0.015, partial η2 = 0.02. On average, workers with children in their households (*M* = 2.38; *SE* = 0.141) evaluated their associations to WFH less positively than workers without children in their households (*M* = 2.80; *SE* = 0.131). Thus, we find statistical support for hypothesis 3 (see Figure 1).

Finally, we investigated whether female workers with children experience WFH less positively than male workers with children in the same household (hypothesis 4). Again, we analyzed the number of positive associations on the subset of workers with children in their households (*n* = 131) and tested whether the scores were higher for female workers. Also here, homogeneity of variances was achieved for both groups assessed by Levene’s test for equality of variances (*p* = 0.188). We controlled for age (*F* (1, 126) = 0.98, *p* = 0.325), actual working hours (*F* (1, 126) = 3.340, *p* = 0.070) and leadership role (*F* (1, 126) = 0.419, *p* = 0.519). Overall, female workers with children in the same household (*M* = 2.52; *SE* = 0.25) evaluated their associations to WFH similarly to male workers with children in their households (*M* = 2.27; *SE* = 0.18). We maintain the null hypothesis (*F* (1, 126) = 0.59, *p* = 0.443).

### Workers’ representations of working from home emerging from content analysis of free association data

We have demonstrated earlier that the presence of children resulted in less positive associations towards WFH (see hypothesis 3). To better understand the underlying reasons, we compared the content expressed in participants’ associations towards WFH between all four groups (male / female; children in the same household / no children in the same household). Our main interest was an understanding about how the content associated with “working from home” characterizes each of the four groups rather than a general exploration of commonalities among semantic categories.

A detailed inspection and screening of our dataset in terms of over-representations (a semantic category being significantly over-represented) revealed differences of the frequency of associations between the four groups (see Tables 2, 3). We summarize here the differences between the four groups (gender x children in household):

**TABLE 2:** Contingency table with superordinate categories.

Gender		Women		Men		
Household		With children	No children	With children	No children	Total
*χ2*(27, 1168) = 53.82, *p* = 0.002						

Meaning of “working from home” and COVID-19	Count	6	11	9	7	33
	%	18.2%	33.3%	27.3%	21.2%	100.0%
	Adj. residual	0.2	0.4	−0.1	−0.5	
Negative attitudes	Count	6	5	6	5	22
	%	27.3%	22.7%	27.3%	22.7%	100.0%
	Adj. residual	1.4	−0.8	−0.1	−0.3	
Negative consequences for private life or health**	Count	**42**	38	44	23	147
	%	**28.6%**	25.9%	29.9%	15.6%	100.0%
	Adj. residual	**4.2**	−1.2	0.5	−2.8	
Negative consequences for working	Count	7	19	11	11	48
	%	14.6%	39.6%	22.9%	22.9%	100.0%
	Adj. residual	−0.4	1.5	−0.8	−0.4	
Personal experience	Count	18	23	24	25	90
	%	20.0%	25.6%	26.7%	27.8%	100.0%
	Adj. residual	0.9	−1.0	−0.3	0.6	
Positive attitude**	Count	12	24	33	**38**	107
	%	11.2%	22.4%	30.8%	**35.5%**	100.0%
	Adj. residual	−1.6	−1.8	0.6	**2.6**	
Positive consequences for private life or health	Count	37	74	65	58	234
	%	15.8%	31.6%	27.8%	24.8%	100.0%
	Adj. residual	−0.4	0.6	−0.1	−0.2	
Positive consequences for society	Count	6	4	7	8	25
	%	24.0%	16.0%	28.0%	32.0%	100.0%
	Adj. residual	1.0	−1.5	0.0	0.8	
Positive consequences for working**	Count	37	**120**	80	84	321
	%	11.5%	**37.4%**	24.9%	26.2%	100.0%
	Adj. residual	−2.9	**3.4**	−1.5	0.5	
Requirements and conditions*	Count	23	33	**50**	35	141
	%	16.3%	23.4%	**35.5%**	24.8%	100.0%
	Adj. residual	−0.1	−1.8	**2.1**	−0.1	
Total	Count	194	351	329	294	1168
		16.6%	30.1%	28.2%	25.2%	100.0%

Frequencies of associations assigned to the categories by children and gender.

When a category is underrepresented (i.e., adjusted residual < −1.96 at *p* < 0.05 level = *; adj. residual < −2.58 at *p* < 0.01 level = **) or overrepresented (i.e., adj. residual > 1.96 at *p* < 0.05 level = *; adj. residual > 2.58 at *p* < 0.01 level = **) values are indicated in bold.

**TABLE 3:** Contingency table with semantic categories.

Gender		Women		Men		
Household		With children	No children	With children	No children	Total
χ2(27, 1168) = 151.88, *p* < 0.001						

Adaptation	Count	5	8	9	8	30
	%	16.7%	26.7%	30.0%	26.7%	100.0%
	Adj. Res.	0.0	−0.4	0.2	0.2	
Additional burden**	Count	**14**	**2**	**18**	**3**	37
	%	**37.8%**	**5.4%**	**48.6%**	**8.1%**	100.0%
	Adj. Res.	**3.5**	−**3.3**	**2.8**	−**2.4**	
Autonomy	Count	22	43	35	33	133
	%	16.5%	32.3%	26.3%	24.8%	100.0%
	Adj. Res.	0.0	0.6	−0.5	−0.1	
Being home	Count	8	21	14	16	59
	%	13.6%	35.6%	23.7%	27.1%	100.0%
	Adj. Res.	−0.6	1.0	−0.8	0.4	
Communication	Count	11	14	11	15	51
	%	21.6%	27.5%	21.6%	29.4%	100.0%
	Adj. Res.	1.0	−0.4	−1.1	0.7	
Complicated	Count	3	3	3	5	14
	%	21.4%	21.4%	21.4%	35.7%	100.0%
	Adj. Res.	0.5	-0.7	−0.6	0.9	
Concentration**	Count	**4**	**41**	16	19	80
	%	**5.0%**	**51.3%**	20.0%	23.8%	100.0%
	Adj. Res.	−**2.9**	**4.3**	−1.7	−0.3	
COVID-19	Count	3	6	5	4	18
	%	16.7%	33.3%	27.8%	22.2%	100.0%
	Adj. Res.	0.0	0.3	0.0	−0.3	
Definition	Count	3	5	4	3	15
	%	20.0%	33.3%	26.7%	20.0%	100.0%
	Adj. Res.	0.4	0.3	−0.1	−0.5	
Distraction	Count	3	5	3	3	14
	%	21.4%	35.7%	21.4%	21.4%	100.0%
	Adj. Res.	0.5	0.5	−0.6	−0.3	
Efficiency*	Count	**3**	15	15	16	49
	%	**6.1%**	30.6%	30.6%	32.7%	100.0%
	Adj. Res.	−**2.0**	0.1	0.4	1.2	
Environmentally friendly	Count	6	4	7	8	25
	%	24.0%	16.0%	28.0%	32.0%	100.0%
	Adj. Res.	1.0	-1.5	0.0	0.8	
Exhausting*	Count	**6**	3	2	5	16
	%	**37.5%**	18.8%	12.5%	31.3%	100.0%
	Adj. Res.	**2.3**	−1.0	−1.4	0.6	
Experience	Count	2	1	4	2	9
	%	22.2%	11.1%	44.4%	22.2%	100.0%
	Adj. Res.	0.5	−1.2	1.1	−0.2	
It does not work	Count	3	2	3	0	8
	%	37.5%	25.0%	37.5%	0.0%	100.0%
	Adj. Res.	1.6	−0.3	0.6	−1.6	
It works	Count	11	20	25	26	82
	%	13.4%	24.4%	30.5%	31.7%	100.0%
	Adj. Res.	−0.8	−1.2	0.5	1.4	
Lack of good work space	Count	4	11	6	7	28
	%	14.3%	39.3%	21.4%	25.0%	100.0%
	Adj. Res.	−0.3	1.1	−0.8	0.0	
Less stress	Count	10	17	10	18	55
	%	18.2%	30.9%	18.2%	32.7%	100.0%
	Adj. Res.	0.3	0.1	−1.7	1.3	
Longer hours and blurred boundaries*	Count	**12**	13	9	**4**	38
	%	**31.6%**	34.2%	23.7%	**10.5%**	100.0%
	Adj. Res.	**2.5**	0.6	−0.6	**−2.1**	
No commuting	Count	15	36	27	24	102
	%	14.7%	35.3%	26.5%	23.5%	100.0%
	Adj. Res.	−0.5	1.2	−0.4	−0.4	
Not always possible**	Count	1	3	**14**	**0**	18
	%	5.6%	16.7%	**77.8%**	**0.0%**	100.0%
	Adj. Res.	−1.3	−1.2	**4.7**	−**2.5**	
Personal preferences	Count	5	13	12	11	41
	%	12.2%	31.7%	29.3%	26.8%	100.0%
	Adj. Res.	−0.8	0.2	0.2	0.2	
Practical**	Count	1	4	8	**12**	25
	%	4.0%	16.0%	32.0%	**48.0%**	100.0%
	Adj. Res.	−1.7	−1.5	0.4	**2.7**	
Reduced productivity	Count	0	3	2	1	6
	%	0.0%	50.0%	33.3%	16.7%	100.0%
	Adj. Res.	−1.1	1.1	0.3	−0.5	
Self-regulation*	Count	4	8	**16**	7	35
	%	11.4%	22.9%	**45.7%**	20.0%	100.0%
	Adj. Res.	−0.8	−0.9	**2.3**	−0.7	
Social isolation	Count	10	20	15	11	56
	%	17.9%	35.7%	26.8%	19.6%	100.0%
	Adj. Res.	0.3	0.9	−0.2	-1.0	
Technical infrastructure/equipment*	Count	**18**	s15	14	21	68
	%	**26.5%**	22.1%	20.6%	30.9%	100.0%
	Adj. Res.	**2.3**	−1.5	−1.4	1.1	
Trust vs. control*	Count	**0**	7	6	7	20
	%	**0.0%**	35.0%	30.0%	35.0%	100.0%
	Adj. Res.	−**2.0**	0.5	0.2	1.0	
Work-life balance*	Count	7	8	**16**	5	36
	%	19.4%	22.2%	**44.4%**	13.9%	100.0%
	Adj. Res.	0.5	−1.0	**2.2**	−1.6	
Total	Count	194	351	329	294	1168
		16.6%	30.1%	28.2%	25.2%	100.0%

Frequencies of associations assigned to the categories by children and gender.

When a category is underrepresented (i.e., adjusted residual < −1.96 at *p* < .05 level = *; adj. residual < −2.58 at *p* < 0 .01 level = **) or overrepresented (adj. residual > 1.96 at *p* < 0.05 level = *; adj. residual > 2.58 at *p* < 0 .01 level = **) values are indicated in bold.

*Female workers with children in their household* associated on the superordinate level significantly more often terms that are related to aspects that can be subsumed under “negative consequences for private life or health” (res_adj_ = 4.2, *p* < 0.01) compared to the other three groups (see Table 2). In more details (see Table 3), associations encompassed terms such as “exhaustion” (res_adj_ = 2.3, *p* < 0.05), “longer hours and blurred boundaries” (res_adj_ = 2.5, *p* < 0.01) or “additional burden” (res_adj_ = 3.5, *p* < 0.01). Female workers with children were also more concerned with “technical infrastructure/equipment” (res_adj_ = 2.3, *p* < 0.05) and associated less often terms such as “efficiency” (res_adj_ = −2.0, *p* < 0.05), “(better) concentration” (res_adj_ = −2.9, *p* < 0.01), or mentioned less often issues about “trust vs. control” (res_adj_ = −2.0, *p* < 0.05).

*Female workers without children in their household* mentioned significantly more associations that relate to “positive consequences for working” (res_adj_ = 3.4, *p* < 0.01) compared to the other groups. In more detail, associations encompassing “(better) concentration” (res_adj_ = 4.3, *p* < 0.01) were mentioned more often than in any other group and associations about “additional burden” (res_adj_ = −3.3, *p* < 0.01) were mentioned less frequently than from (female and male) workers with children.

*Male workers with children in the household* were significantly more concerned with “requirements and conditions” (res_adj_ = 2.1, *p* < .05) of WFH than the other groups. In this superordinate category, categories such as “not always possible” (res_adj_ = 4.7, *p* < 0.01), requires “(more) self-regulation” (res_adj_ = 4.2, *p* < 0.01) or “additional burden” (res_adj_ = 4.2, *p* < 0.01) are subsumed. They also noted on the positive side that it helps to establish a positive “work-life balance” (res_adj_ = 2.3, *p* < 0.05).

Finally, *male workers without children* revealed more “positive attitudes” to WFH (res_adj_ = 2.6, *p* < 0.01) on the superordinate level than any other group. They perceived WFH as “practical” (res_adj_ = 2.7, *p* < 0.01) and expressed significantly less frequently associations concerning “longer hours and blurred boundaries” (res_adj_ = −2.1, *p* < 0.05) to WFH or that it is “not always possible” (res_adj_ = −2.5, *p* < 0.05). Associations that related to an “additional burden” (res_adj_ = −2.4, *p* < 0.05) were less frequent than from workers with children.

To obtain a synopsis on the co-occurrences of the categories of the variables reported in this study, we carried out a categorical exploratory analysis. Multiple Correspondence Analysis (MCA) is an explorative geometric technique (Hjellbrekke, 2018) for the analysis of co-occurrences reported in a contingency table of multivariate categorical data. Based on calculated chi-square distances between variables, data were projected into a geometric representation in which the distances between variables are directly interpretable in terms of relatedness / non-relatedness. We opted for the use of Categorical Principal Component Analysis (Linting et al., 2007; CATPCA, Grouping Method; SPSS 27), an extension of MCA, to include ordinal variables in the analysis such as workers’ attitudes towards WFH as expressed in their evaluation of associations. Alike MCA, CATPCA, allows for the distinct use of active and supplementary variables whereby the masses (weighted frequencies) of the supplementary variables are not taken into account in the definition of the dimensions. Participants’ categorized associations were projected as non-active supplementary variables together with socio-demographic variables (see Table 1). The reduced number of active variables in the analysis enables a focused interpretation of the dimensions with respect to our research interest, i.e., to understand workers’ representations about WFH based on their gender, children in the household and attitudes towards WFH. Not all our variables of interest were nominal or multiple nominal such as participants’ attitudes (ordinal, 3 nodes), size of their organization (ordinal, 4 nodes), and team size (ordinal, 4 nodes). All other supplementary variables had a nominal or multiple nominal scale (“education,” “industry,” “position”). Consequently, only a relatively low percentage of variables’ variances was accounted for (see Table 4).

**TABLE 4:** CATPCA dimension discrimination measures.

	CATPCA Dimension		
	1	2	*M*
Children in household	**0.579**	0.000	**0.289**
Gender	**0.345**	**0.488**	**0.416**
Attitudes towards WFH	**0.351**	**0.467**	**0.409**
Evaluation*^a^*	0.093	0.149	0.121
Highest educational degree***^a^***	0.022	0.006	0.014
What industry are you in?***^a^***	0.083	0.012	0.048
Leadership position?***^a^***	0.010	0.035	0.023
Size of the organization***^a^***	0.004	0.054	0.029
Number of persons in teams***^a^***	0.006	0.024	0.015
10 superordinate semantic categories***^a^***	0.068	0.136	0.102
29 semantic categories***^a^***	0.105	0.163	0.134
Active Total	1.275	0.955	1.115
% of variance	39.8%	29.8%	1.601

^a^Supplementary variable (masses not considered); in bold: active variables.

**FIGURE 2:**
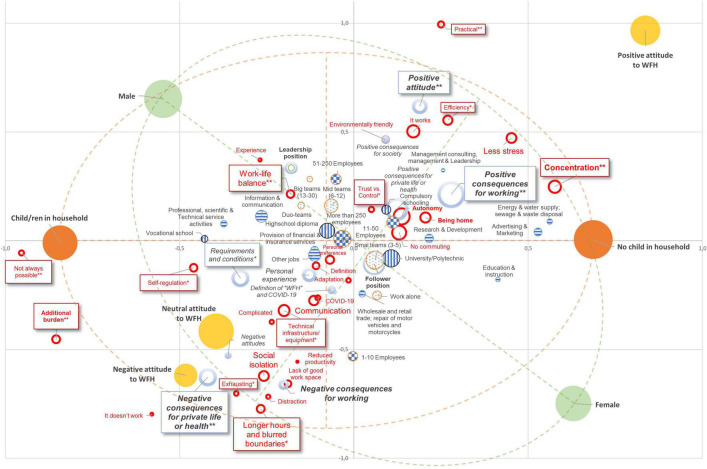
CATPCA-biplot: dimension 1: 39.8%; dimension 2: 29.8%. Fully colored circles represent the seven categories of the three main variables of our analysis (*gender*: “male”/“female” in green, *children in household*: “child/ren in household”/“No child in household” in orange, *evaluations of associations*: “positive attitudes towards WFH”/“neutral attitudes towards WFH”/“negative attitudes towards WFH” in yellow). The size of the circles represents the frequency (mass) of each category (the bigger the circle, the more frequent the category). The proximity of circles may be directly interpreted in terms of their correspondence to the three main variables. Italic letters indicate the superordinate categories. Red fonts indicate the 29 categories. Category labels with framing indicate their significant over- and/or under-representations as reported in Table 2 (superordinate categories) and Table 3 (semantic categories). To ease the interpretation, we added two orthogonal axes (dotted lines) aligned with (1) the poles of gender variable in green and (2) the poles of household condition in orange. The circles without filling colors (education = horicontal lines; number or persons in teams: dots; industry: vertical lines; size of the organization: squared); represent the categories of supplementary variables added to CATPCA. Their mass is not considered in the analysis (Hjellbrekke, 2018) and facilitates the interpretation of the bidimensional Euclidean space.

Figure 2 provides a synopsis of how the different variables and their categories link to each other. The interpretation of this biplot is aided by inspecting the adjusted standard residuals of each semantic category (Agresti, 2002) reported in Tables 2, 3. Dimension 1 (39.8%) expressed 100% of the variable “children in the same household” with the category “no child in household” being projected on the positive axis and “child/ren in household” on the negative axis of dimension 1. Our second principal variable - gender - had “male” projected on the negative axis of dimension 1 and on the positive axis of dimension 2; “female” was projected symmetrically on the other side of the variable’s barycenter. Finally, almost orthogonally, workers with positive attitudes towards WFH resulted in the positive poles of both dimensions and negative attitudes are shown on the diametric pole. The cloud of semantic categories resulted along the attitudes spanning from more critical content (e.g., “additional burden,” “longer hours and blurred boundaries,” “exhausting,” etc.) in the negative quadrant of the biplot (lower left side of Figure 2) to more positive content favoring WFH (“practical,” “less stress”; “(better) concentration,” “it works”) that was projected in the positive quadrant of the biplot (upper right side of Figure 2).

## Discussion

The overall aim of this study was to investigate whether *working from home* (WFH) during the pandemic was a different experience for female and male workers, in particular when they had to take care of dependent children in the same household. Based on social role theory as well as empirical studies from the pandemic (e.g., Derndorfer et al., 2021), we assumed increased gender inequality particularly among mothers working from home (e.g., Yerkes et al., 2020). To gain more insights about gendered dynamics, we employed an exploratory approach and investigated remote workers’ representations about WFH. Our findings revealed that gender and children in the household need to be considered to understand representations about WFH by workers who lived with their intimate partners in the same household. Although the results from the quantitative analysis indicate that having children per se did not make mothers and fathers interrupt work for non-work behaviors more often than workers without children, both mothers and fathers with children experienced WFH during the pandemic as less positively than women and men without children. Parents particularly emphasized the additional burden due to childcare responsibilities. Interestingly, when there were no children in the household, women seemed to benefit mostly from more focused work at home whereas men considered WFH mostly as efficient and practical. In the following, we discuss our findings in more detail.

*No boundary management strategy based on children and gender.* Our results revealed no significant differences of non-work interrupting work behaviors between workers who have children and those without children (hypothesis 1), neither did we find differences between mothers and fathers (hypothesis 2). Children in the same household did not significantly influence non-work interrupting work behaviors. However, WFH was rarely a voluntary choice during the lockdown and workers were highly affected by other persons living in the same household (Langenkamp et al., 2022). Thus, many workers had limited control to adjust their non-work sphere according to their needs (e.g., being undisturbed during working hours at home). It can be assumed that different strategies between workers with children and those without are more likely when working from home is perceived as a choice rather than an exogenous demand, and the interruption is perceived as internally controllable. The content analysis of the associations shows that for mothers, exhaustion and blurred boundaries were more prevalent than for fathers. Research shows that women are not always able to enact their *preferred* boundary management strategy (Ammons, 2013), and feel more in need for control over boundary permeability than men due to the many demands and spillover effects from the other sphere (Straub et al., 2019). As such, particularly mothers might be eager to control the boundaries and segment their work from the non-work sphere. However, it is important to note that associations related to more autonomy and flexibility were equally balanced across the four groups (women and men with / without children).

*Childcare during WFH was a challenge for both mothers and fathers.* Analyzing workers’ free verbal associations towards WFH helps to deconstruct workers’ underlying challenges when providing childcare during WFH. In line with our expectations the results show that workers evaluated WFH more positively when there were no children in the same household (hypothesis 3). Furthermore, the analysis revealed no differences between mothers and fathers (hypothesis 4). Although WFH is traditionally seen as a way to reconcile work and non-work, there are several challenges to cope with for both mothers and fathers. Thus, despite several studies showing that mothers had to take a larger share of this additional childcare responsibilities during the pandemic (e.g., Chung et al., 2021; Derndorfer et al., 2021; Dunatchik et al., 2021), fathers experienced the hassle to cope with the additional child caring demands at a similar extent. On the positive side, both mothers and fathers acknowledged the positive effects of WFH despite the increased challenge to juggle the demands from both spheres. Our findings show that WFH allows fathers to also contribute more to family life. This is in line with a recent Canadian study (Shafer et al., 2020), which explains fathers’ involvement with a needs-exposure hypothesis suggesting that fathers’ contribution positively shifts towards a more egalitarian divide when the immediate need arises.

*WFH as potential work-family facilitation for fathers.* Despite the mentioned positive effects of WFH for both mothers and fathers, mothers more strongly experienced distress and exhaustion, less concentration and longer working hours compared to fathers. Also, mothers reported *less* often that WFH is efficient and more often associated terms related to necessary infrastructure / equipment suggesting a potential lack thereof. Thus, mothers struggled with meeting demands from both spheres potentially impairing their work. Fathers were more likely to stress the need for self-regulation and discipline while working from home. Such an agency and self-optimization perspective implies the discretion to choose and control one’s work and non-work sphere and the boundaries and interruptions between them (and hence make it easier to integrate demands from both spheres). In line with that, mothers more often mentioned terms that revealed a perceived incompatibility between the work and non-work sphere, while fathers’ representations referred to work-life/family facilitation of WFH, which corroborates pre-pandemic studies (e.g., Halford, 2006; Borgkvist et al., 2018). Women are more likely to struggle with the combination of having a family and a highly demanding job, whereas for men work-family balance is less affected by this situation (Kinnunen et al., 2004). Findings about mothers who reduced work hours instead of fathers further resemble this argument (Chung et al., 2021). While WFH indeed might be a chance for families to distribute family demands more evenly and make fathers more easily engage in family life (Derndorfer et al., 2021), there is otherwise the risk that women are left with childcare demands at the expense of the work sphere. This highlights the importance that fathers are provided with WFH, and then share the non-work demands equally with their cohabitating partners. However, our study suggests that fathers have more difficulties to gain access to WFH than mothers. Thus, not only fathers and mothers themselves have to actively rethink their current practices of division of labor in their households, but organizations need to enable them to do so. Although prevailing social norms seem to have already partly shifted towards greater equality and more acceptance of fathers’ parental leave (Lott and Klenner, 2018), the male worker image still does not encompass child caring duties. To reach more equality here, policies need to be formulated and counteract the practice that organizations still hesitate to let fathers work from home. Organizations need to provide flexible work arrangements *and* re-frame their WFH arrangements as facilitation of work and life demands beyond childcare (e.g., include voluntary work or care work for elderly or the community). Only when WFH is no more stigmatized as a tool for mothers with lower career aspirations, it can indeed facilitate “work-life balance” for all groups. Then, it has the potential to also lose the stigma of lower productivity and decrease the distress of boundary blurring in WFH.

*The under-researched beneficiaries of WFH: women without children.* Comparing the semantic content of the associations between women and men *without* children also suggests gendered representations about WFH in the work sphere. Women without children particularly mentioned that WFH provides concentration for their work whereas men without children more often considered WFH as practical and simple, which enables the reduction of working hours (although time saved for commuting was mentioned equally frequent within all groups). These findings might suggest that women mostly consider WFH as beneficial for their work whereas men consider the increase for their quality of life. Following this, we argue that gender-specific barriers in the gender segregated work sphere need to be tackled. For example, studies from academia suggest that women are more likely to be asked to volunteer and accept tasks with low promotability such as being on committees instead of working on research papers (Babcock et al., 2017). We assume that this gender effect is also prevalent in other industries and interpret our finding in the way that women’s potential for more concentration when working from home is due to less (non-promotable) ad-hoc requests from colleagues and supervisors when working out of sight. Therefore, it is important that organizations are aware of potential gender disparities in the way work tasks are assigned.

To sum up, not only women with children profit from WFH, but also women without children – suggesting again to reframe WFH as going beyond a reconciliation of work and childcare. This is particularly important as disparities in the paid work sphere are highly interwoven with the distribution of unpaid work in couple households, and may also affect gendered WFH experiences. We can assume that “gender contracts” exist in both egalitarian and non-egalitarian Austrian couples, where issues like unpaid work such as childcare or housework are negotiated by the partners (Haandrikman et al., 2019). Although it is likely in Austria that women in households without children might have a more equal distribution of income and working hours, this usually changes once there are children in the family resulting in a lack of “bargaining power” for women at home (Derndorfer et al., 2021). Thus, women without children might have had more bargaining power over unpaid work in their household and therefore focused on work-related benefits of WFH in their representations. In line with that, a decrease in productivity when working from home is more unlikely for women in partnerships without children (Weitzer et al., 2021). Thus, again, organizations can support female workers and limit the motherhood penalty by giving fathers easier access to WFH and flexible work arrangements. However, this will only be beneficial for women with children if fathers then indeed engage in deep-care tasks and other demands from the non-work sphere.

### Future research - Gendered work/family conflict and interruptions when working from home?

Our findings raise many theoretically important questions about gendered work-life/family conflict that need to be further investigated in future studies. For example, work-family conflict often stems from a perceived incompatibility of different role demands. Due to social role pressures, male workers with care obligations for dependent children might perceive the incompatibility less strongly than female workers. This suggests that not necessarily all interruptions when working from home (in particular for men) result in perceived work-family conflict and require a specific boundary management strategy as response. While some interruptions may indeed be intrusive and require a role transition (e.g., discussing an emotional topic with a family member over the phone during work time), others might hardly affect the work sphere (e.g., arranging a doctor’s appointment). We call for more knowledge about the content of the interruptions (Hunter et al., 2019; Horvath et al., 2021). Future research needs to shed light on the question whether women and men are affected distinctively by specific interruptions from the non-work sphere. Exploring the content of interruptions helps to better understand how women and men can benefit best from the possibility of WFH.

Also, a different impact of WFH for women and men on their careers is likely as mothers are still considered the predominant caretakers for the children. Although social norms are shifting (Lott and Klenner, 2018), our findings corroborate earlier research and show that it is still a long way to go for gender equality. One important step in this direction would be to re-frame WFH in the (Austrian) public discourse and consider it as means of work/non-work facilitation rather than the sole focus on reconcilability between work and childcare. Non-work demands, interests and care needs might go beyond childcare and can also include caring for parents or engaging in community work. This would also have implications on workers’ careers as research shows managers’ attributions why followers use WFH also affects employees’ career success (Leslie et al., 2012). Finally, our findings imply the need to establish institutional requirements for fathers. Policy-makers in Austria and employers need to take into consideration that fathers’ opportunity for WFH benefits mothers also by reducing their role conflicts and allowing them to prioritize work more. Thus, future research needs to address and overcome organizational and social barriers for men taking family responsibilities. Organizational measures such as non-standard working schedules for all workers and more awareness about the non-work demands for workers beyond childcare can help to tackle persistent gender norms and role pressures arising from the ideal worker image.

## Limitations

It is important to note limitations of this study. For example, the number and age of children are likely to affect experiences about WFH (Schieman et al., 2021). Unfortunately, we did not collect enough data to systematically compare the age of the children. Moreover, we did not obtain any information about the partner’s job situation. Although we conducted an accompanying survey aimed at the partners, we could not gain enough data. Therefore, we were not able to control whether a partner’s support in childcare and other variables such as the partner’s working hours affected associations and interruptions while working from home (Russo et al., 2018).

With regard to generalizability, we suggest that the Austrian context with traditional gender norms and distribution of unpaid vs. paid work might have shaped our results. Also, women with children were slightly underrepresented in our sample, while men with children were overrepresented. As mothers seemed to be more affected by additional family demands, there is potential bias of our results. A potential cause for the underrepresentation of women with children might be that they did not have time to fill it in (and supposedly strengthening the gender effect) as usually women are more likely than men to participate in surveys (Søgaard et al., 2004). For data collection we used the members’ list from the Chamber of Labor of Lower Austria to include participants from a diverse range of work contexts, but a rather selective family context such as remote workers who live with their intimate partners in the same household. Despite our aim to reach a wide sample, we assume that our results have limited applicability for singles and single parents, as social isolation was found as a major stressor during the lockdown (Langenkamp et al., 2022).

## Conclusion

Gender(ed) practices at work and in families are often non-reflective, and non-intentional routines (Martin, 2003). Working conditions and the nature and extent of (ad hoc) demands and spillovers from the other sphere may be different for women and men (Straub et al., 2019), leading to different (unconscious) routines in dealing with interruptions and different needs for managing boundaries. Existing work arrangements in the pandemic made evident that work and non-work spheres have indeed always impacted each other, yet that the demands from the spheres are still often incompatible for many workers (Kossek et al., 2021). Employing the free verbal association technique (Joffe and Elsey, 2014) enabled us to uncover positive as well as negative aspects of WFH. Investigating the role of children and gender, our study shows that WFH is particularly impacted by children in the same household. Mothers engaging in WFH seemed to find the juggling between spheres most challenging whereas fathers particularly mentioned how it enables work-family facilitation. Most interestingly, gendered dynamics seem also be in place when there are no children in the same household. Women without children benefit mostly from more concentration when working from home, and men without children appreciate WFH as being practical and efficient. Thus, we conclude that organizations need to be aware about workers’ different (family) contexts and WFH needs to be re-framed as opportunity for job-crafting based on personal needs and preferences (Wessels et al., 2019) for all groups of workers rather than promoting a narrow view of WFH as reconciliation between work and non-work demands for working mothers (Schmidt, 2021).

## Data availability statement

The raw data supporting the conclusions of this article will be made available by the authors, without undue reservation.

## Ethics statement

Ethical review and approval was not required for the study on human participants in accordance with the local legislation and institutional requirements. The patients/participants provided their written informed consent to participate in this study.

## Author contributions

MH-T: planning and execution of the study, categorizing associations, interpreting results, and writing – original draft preparation and review and editing. EZ: interpreting results, and writing – original draft preparation and review and editing. TS: analyzing data and interpreting and writing-up results. All authors contributed to the article and approved the submitted version.

## Supplementary material

Supplementary Table 1 Description of the 29 semantic categories.

Supplementary Table 2 Dimensions and component loadings for the variables and categories included in the CATPCA and represented in the biplot of Figure 2.
