# Supplementary material for: Remote workers’ free associations with working from home during the COVID-19 pandemic in Austria: The interaction between children and gender
Source: Front Psychol. 2022 Aug 4;13:859020. doi: 10.3389/fpsyg.2022.859020 (PMC9391219; doi:10.3389/fpsyg.2022.859020)
Supplement: Supplementary Table 1 — Description of the 29 semantic categories. [file Data_Sheet_1.docx]

**Supplementary Table 1: Description of the 29 semantic categories**

| **Categories** | | **Examples** | **Description** |
| --- | --- | --- | --- |
| **1. Meaning of "working from home" and COVID-19** | | | |
| Definition | | Homeoffice; teleworking | “Working from home” is translated into one's own language. The term “home office” is commonly used (in German) |
| COVID-19 | | Corona; short time; labor law | Associations with corona-related changes are addressed and feelings regarding the pandemic (e.g., helpless, social). Reference to labor law and related changes are also included here. |
| **2. Personal experience** | | | |
| Adaptation | | New; no routine; use of own resources; saves money; no costs for commuting | A new and unfamiliar situation requires getting used to and adapting to the new working life from home. Terms describe the new, unfamiliar situation that was quickly introduced. Regarding costs, there are both aspects of savings (e.g., fuel), but also additional costs (e.g., electricity). |
| Experience | | I am used to it; normal; not new | Working from home is nothing new and was a common way of working even before the pandemic. |
| Communication | | Videoconferencing; availability; skype; MS teams; online; no direct contact with colleagues | Communication is changing: virtual and telephone meetings and conferences are mentioned frequently. Contact with colleagues and availability are perceived differently. Some feel that they can be reached easily, while others mention misunderstandings and conflicts. This category relates to communication within working hours (or it is not clear whether within or outside of working hours). |
| **3. Requirements and conditions** | | | |
| Not always possible | | Not possible; would be preferable; from time to time; emergency | Not all people and professional groups can carry out their activities from home. However, encouragement for WFH is not the only and permanent solution with the desire for a mixed form that brings variety. |
| Technical infrastructure/ equipment | | Laptop; telephone; internet | Requirements (internet connection, but also technical devices, office equipment and work space) for WFH. |
| Trust vs. Control | | Trust; control; responsibility | Trust is an important aspect for functioning of WFH. But mistrust and control are still an issue. Workers are given more personal responsibility. |
| Self-regulation | | Self-organization important; motivation hard; organizing; planning | WFH requires organization and demarcation. This also requires a lot of discipline and planning. Switching off and delimiting (mentioned alone) also fall into this category. |
| **4. Positive attitude** | | | |
| Practical | | Practical; easy; possible; useful | Positive attributions such as practical, simple, sensible and easy show that WFH is rated positively. More detailed explanations are not given. |
| It works | | Motivating; super; nice; works good | Descriptions that express that WFH is possible, gives energy and motivation and is seen as effective. However, there are no more detailed explanations which aspects are particularly valued. Also, it is seen as contemporary and modern. A working model of the future. |
| **5. Positive consequences for working** | | | |
| Autonomy | | Flexible time management; self-determined | Once people are flexible regarding work space, temporal flexibility is also likely. Many associations concern flexibility, flexible time management and the autonomy and freedom often associated with it. |
| Being home | | Cozy; comfortable; familiar atmosphere; cat | Associations that indicate that it is cozy at home and you can feel comfortable. The atmosphere is familiar and animals, gardens, etc. are present. |
| Concentration | | Focused work; undisturbed; less disturbances from colleagues | To be able to work in a focused manner, you need quiet and concentration. Interruptions and disturbances from colleagues are less frequent. |
| Efficiency | | Efficient; productivity; more performance | Terms such as efficient and productive show that the output and performance is also seen as positive. |
| **6. Positive consequences for private life or health** | | | |
| No commuting | | no commuting; saves time; time; more spare time; no traffic jam | By working from home, commuting to the workplace is not necessary, which saves travel time and avoids traffic jams. |
| Personal preferences | | Sleeping in; comfortable work clothes; freshly cooked | WFH makes it possible to work more in line with one's own preferences. For example, you can get up later and thus it provides more sleep, it enables cooking fresh food and the clothes can be more casual and comfortable. |
| Less stress | | Relaxed; increases quality of life; free of stress | People feel less stressed and more relaxed, which contributes to a higher quality of life. With WFH, there is no hustle and bustle. |
| Work-life balance | | Better work-life balance; family friendly; more time for family | WFH is related to work-life balance. It facilitates compatibility and means a plus in time together with the family. |
| **7. Positive consequences for society** | | | |
| Environ-mentally friendly | | Less traffic; climate friendly; environmentally friendly | Since the need to travel by car has been eliminated and thus there is less traffic, positive effects for the environment are mentioned. |
| **8. Negative attitudes** | | | |
| Complicated | | Hard; tedious; everything takes longer; complicated; annoying | Due to the lack of exchange in the office, WFH is perceived as difficult, tedious and restless. It lacks structure and everything takes longer. |
| It does not work | | I do not like it; problems; challenge; no | Aversive attitudes without justification. WFH is considered as impossible and is experienced as unsatisfactory that represents a challenge and "does not have to be". |
| **9. Negative consequences for working** | | | |
| Lack of good work space | Lack of infrastructure; it is not ergonomic; small desk; less space | | Lack of infrastructure and equipment to ensure a pleasant and functioning working from home. The lack of space is often mentioned, which means that people must work at the dining table, i.e., from an ergonomic perspective sub-optimal. Consequences are back pain or headache. |
| Distraction | Distraction; too many disturbances | | For some, however, there are also distractions and disturbances at home that impair concentration. |
| Reduced productivity | Less productive; inefficient | | Few associations describe "reduced productivity" |
| **10. Negative consequences for private life or health** | | | |
| Social isolation | | Isolation; loneliness; boring; the informal communication is missing | The lack of contact with colleagues and customers promotes social isolation, and in many cases, it leads to a feeling of loneliness. Terms like boring also fall into this category. |
| Exhausting | | Stressful; exhausting; no relaxation at home | WFH is also exhausting and leads to more stress. If only stress is mentioned, then it is assumed that this is perceived more strongly. |
| Longer hours and blurred boundaries | | Longer working hours; over work; no break; work around the clock; detachment problems | This often results in longer working hours than before. A strict separation of work and private life is more difficult to do because you are constantly available. This leads to demarcation problems and fewer breaks. Work is also done outside standard working hours (e.g., evenings, weekends). |
| Additional burden | | Stress because of family; children; children at home; household; house work | The additional housework and the care of the children during working hours ensure a higher load when working from home. |

**Supplementary Table 2: CATPCA-dimension loadings of all the variables considered in the CATPCA- joint biplot (see Figure 2)**

|  |  | Centroid Coordinates | |
| --- | --- | --- | --- |
| Variable | Category | Dimension 1 | Dimension 2 |
| Children in Household | children in household | -0.843 | -0.009 |
|  | no children in household | 0.686 | 0.005 |
| Gender | female | 0.629 | -0.747 |
|  | male | -0.547 | 0.651 |
| Evaluation of Associations | negative attitude to WFH | -0.482 | -0.618 |
|  | neutral attitude to WFH | -0.395 | -0.415 |
|  | positive attitude to WFH | 0.836 | 0.966 |
| Highest educational degree | compulsory schooling | 0.092 | 0.142 |
|  | vocational school | -0.428 | 0.008 |
|  | high school diploma | -0.076 | 0.046 |
|  | university/polytechnic | 0.107 | -0.080 |
| What industry are you in? | energy & water supply; sewage & waste disposal | 0.561 | 0.088 |
|  | information & communication | -0.265 | 0.112 |
|  | Education & Instruction | 0.413 | -0.178 |
|  | Research & Development | 0.216 | 0.010 |
|  | Advertising & Marketing | 0.528 | 0.039 |
|  | Wholesale and retail trade; repair of motor vehicles and motorcycles | 0.023 | -0.243 |
|  | Provision of financial & insurance services | -0.112 | -0.044 |
|  | Professional. scientific & technical service activities | -0.373 | 0.077 |
|  | Management consulting, management & leadership | 0.256 | 0.323 |
|  | Other jobs | -0.112 | -0.065 |
| Leadership position? | leadership position | -0.179 | 0.334 |
|  | follower position | 0.058 | -0.105 |
| Size of the organization | 1-10 workers | -0.003 | -0.530 |
|  | 11-50 workers | 0.111 | 0.084 |
|  | 51-250 workers | -0.051 | 0.282 |
|  | more than 250 workers | -0.033 | 0.009 |
| Number of persons in teams | work alone | 0.066 | -0.250 |
|  | duo-teams | -0.151 | 0.161 |
|  | small teams (3-5) | 0.060 | -0.088 |
|  | mid teams (6-12) | -0.065 | 0.160 |
|  | big teams (13-30) | -0.126 | 0.283 |
| 29 semantic Categories | adaptation | -0.108 | -0.115 |
|  | additional burden** | -0.854 | -0.452 |
|  | autonomy | 0.136 | 0.111 |
|  | being home | 0.205 | 0.106 |
|  | communication | -0.114 | -0.275 |
|  | complicated | -0.235 | -0.373 |
|  | concentration** | 0.576 | 0.248 |
|  | COVID-19 | -0.103 | -0.262 |
|  | definition | -0.015 | -0.182 |
|  | distraction | -0.245 | -0.716 |
|  | efficiency* | 0.269 | 0.554 |
|  | environmentally friendly | 0.090 | 0.464 |
|  | exhausting* | -0.337 | -0.701 |
|  | experience | -0.269 | 0.371 |
|  | it does not work | -0.579 | -0.796 |
|  | it works | 0.170 | 0.502 |
|  | lack of good work space | -0.188 | -0.656 |
|  | less stress | 0.452 | 0.472 |
|  | longer hours and blurred boundaries* | -0.267 | -0.772 |
|  | no commuting | 0.129 | 0.037 |
|  | not always possible** | -0.953 | -0.056 |
|  | personal preferences | -0.068 | -0.087 |
|  | practical** | 0.249 | 0.994 |
|  | reduced productivity | -0.162 | -0.556 |
|  | self-regulation* | -0.459 | -0.124 |
|  | social isolation | -0.258 | -0.621 |
|  | technical infrastructure/ equipment* | -0.200 | -0.320 |
|  | trust vs. control* | 0.050 | 0.143 |
|  | work-life balance* | -0.182 | 0.214 |
| 10 superordinate categories | definition of "WFH" and COVID-19 | -0.063 | -0.225 |
|  | negative attitudes | -0.360 | -0.527 |
|  | negative consequences for private life or health | -0.419 | -0.626 |
|  | negative consequences for working | -0.202 | -0.661 |
|  | personal experience | -0.128 | -0.157 |
|  | positive attitude | 0.189 | 0.617 |
|  | positive consequences for private life or health | 0.122 | 0.145 |
|  | positive consequences for society | 0.090 | 0.464 |
|  | positive consequences for working | 0.279 | 0.212 |
|  | requirements and conditions | -0.325 | -0.172 |

Note: Dimensions and component loadings for the variables and categories included in the CATPCA and represented in the biplot of Figure 2.
